# In Cortical Neurons HDAC3 Activity Suppresses RD4-Dependent SMRT Export

**DOI:** 10.1371/journal.pone.0021056

**Published:** 2011-06-09

**Authors:** Francesc X. Soriano, Giles E. Hardingham

**Affiliations:** 1 Centre for Integrative Physiology, University of Edinburgh, Edinburgh, United Kingdom; 2 Department of Cell Biology, Faculty of Biology, University of Barcelona, Barcelona, Spain; University of Insubria, Italy

## Abstract

The transcriptional corepressor SMRT controls neuronal responsiveness of several transcription factors and can regulate neuroprotective and neurogenic pathways. SMRT is a multi-domain protein that complexes with HDAC3 as well as being capable of interactions with HDACs 1, 4, 5 and 7. We previously showed that in rat cortical neurons, nuclear localisation of SMRT requires histone deacetylase activity: Inhibition of class I/II HDACs by treatment with trichostatin A (TSA) causes redistribution of SMRT to the cytoplasm, and potentiates the activation of SMRT-repressed nuclear receptors. Here we have sought to identify the HDAC(s) and region(s) of SMRT responsible for anchoring it in the nucleus under normal circumstances and for mediating nuclear export following HDAC inhibition. We show that in rat cortical neurons SMRT export can be triggered by treatment with the class I-preferring HDAC inhibitor valproate and the HDAC2/3-selective inhibitor apicidin, and by HDAC3 knockdown, implicating HDAC3 activity as being required to maintain SMRT in the nucleus. HDAC3 interaction with SMRT's deacetylation activation domain (DAD) is known to be important for activation of HDAC3 deacetylase function. Consistent with a role for HDAC3 activity in promoting SMRT nuclear localization, we found that inactivation of SMRT's DAD by deletion or point mutation triggered partial redistribution of SMRT to the cytoplasm. We also investigated whether other regions of SMRT were involved in mediating nuclear export following HDAC inhibition. TSA- and valproate-induced SMRT export was strongly impaired by deletion of its repression domain-4 (RD4). Furthermore, over-expression of a region of SMRT containing the RD4 region suppressed TSA-induced export of full-length SMRT. Collectively these data support a model whereby SMRT's RD4 region can recruit factors capable of mediating nuclear export of SMRT, but whose function and/or recruitment is suppressed by HDAC3 activity. Furthermore, they underline the fact that HDAC inhibitors can cause reorganization and redistribution of corepressor complexes.

## Introduction

The precise regulation of gene transcription in the nervous system is an integral part of processes that regulate neuronal differentiation, development, long-term plasticity and the prevention of pathological processes. This regulation is achieved in part through a balance between the activity of transcriptional coactivators and corepressors which in turn control gene transcription when recruited to promoter elements via DNA-binding transcription factors.

A key corepressor is Silencing Mediator of Retinoic acid and Thyroid hormone receptors, SMRT (and its close relative N-CoR) [1], [2], [3]. SMRT is a large multi-domain protein which binds to and mediates repression effected by a number of transcription factors including nuclear hormone receptors, C promoter Binding Factor 1 (CBF1), CCAAT/Enhancer Binding Protein C/EBP) ß, Serum Response Factor (SRF), Nuclear Factor Erythroid 2 like-2 (Nrf2) and MADS box transcription Enhancer Factor (MEF) 2 [1], [2], [4], [5]. SMRT exists in a core complex containing Transducin-Beta-Like (TBL) 1, TBL1 Receptor 1, G protein Pathway Suppressor 2 (GPS2) and histone deacetylase (HDAC) 3 [3], [6], [7], [8], [9], [10]. Furthermore, SMRT recruits additional HDACs including the class I HDAC, HDAC1 and the class II HDACs HDAC4, HDAC5 and HDAC7 [1], [2], [3], [11], [12]. HDAC3 is likely to be the primary enzyme responsible for the deacetylase activity in SMRT complexes [3], [13], [14] which interacts with SMRT through SMRT's repression domain 4 (RD4) in the C terminus and its deacetylase activating domain (DAD), on the N terminus [14]. SMRT does not only act as a platform for HDAC3 recruitment, the DAD domain functions as a cofactor for HDAC3 and is necessary and sufficient for HDAC3 enzymatic activation [14].

In the central nervous system SMRT plays a critical role in forebrain development and maintenance of the neural stem cell state [15]. SMRT also can influence neuronal survival: it specifically antagonizes PPARγ coactivator 1α (PGC-1α)-mediated antioxidant effects in neurons [16]. Thus, regulation of SMRT activity can potentially have physiological consequences in the central nervous system. SMRT activity can be regulated in multiple ways. Classically, it gets displaced from nuclear receptors by the presence of the cognate hormone, which causes a conformational change in the receptor, creating the ‘ligand-form’ that causes SMRT to dissociate and a CREB Binding Protein/p300-containing coactivator complex to associate [17]. Other signal pathways can also affect interaction of SMRT with transcription factors, for instance, phosphorylation of SMRT by MAPK MEK1 and MEKK1 inhibits interaction of SMRT with nuclear receptors [18], while SMRT phosphorylation by Casein Kinase II stabilizes the interaction [19]. SMRT stability is also subject to dynamic control. Recruitment of the ubiquitin machinery by TBL and TBLR mediates degradation of SMRT [20], while phosphorylation by Cyclin Dependent Kinase 2 creates a Pin binding site which also targets SMRT for degradation [21].

Nuclear export is another way by which different stimuli can modify the function of transcription factors and cofactors, including SMRT. Under normal conditions SMRT exists in subnuclear domains colocalized with HDACs [2], [22], [23] and this can be disrupted by several signal pathways, leading to SMRT export from the nucleus. Several kinases such as MEK1, MEKK1, AKT or Ikkα phosphorylate SMRT, promoting nuclear export [2], [18], [24], [25]. In neurons, we showed that synaptic activity induces nuclear export of SMRT via a mechanism involving nuclear Ca^2+^-dependent CaM kinase activity as well as the Ras-ERK1/2 pathway [26]. The exact region of SMRT responsible for mediating signal-dependent export has remained elusive: deletion and truncation analysis revealed that no one domain is responsible [16].

HDAC inhibition is emerging as an attractive therapy for a number of neurodegenerative diseases as well as acute disorders such as stroke [27]–[28]. While HDAC inhibition leads to histone hyperacetylation and altered gene transcription as result of this, many transcription factors and cofactors are also substrates for HDACs. Interestingly, the localization of SMRT is itself dependent on HDAC activity. Inhibition of HDAC activity disrupts its localization within subnuclear domains and in neurons we found that this leads to nuclear export [23], [26]. As such, HDAC inhibition not only inhibits enzyme activity, but in doing so causes the relocalization of this key repressor. Thus, there is a strong interdependence between SMRT and HDACs: SMRT is an essential activating cofactor for HDAC3, while HDAC activity is crucial for both the repressive function of SMRT and for its nuclear localization. Here we have further investigated the basis of SMRT export following HDAC inhibition, both in terms of the specific HDAC involved, as well as the region(s) of SMRT responsible for mediating export. We provide evidence that SMRT nuclear localization specifically requires Class I HDAC activity, likely to be HDAC3. Furthermore we show that full nuclear localization requires the HDAC3-activating DAD of SMRT. We also demonstrate that export following HDAC inhibition is mediated by SMRT's repression domain 4 (RD4) region and is therefore mechanistically different from export induced by synaptic activity.

## Materials and Methods

### Neuronal cultures and stimulations

All animal tissue was obtained by schedule 1 methods in accordance with the Animals (Scientific Procedures) Act 1986 and with the agreement of the University of Edinburgh Ethical Review Committee for which a specific project licence is not required. Cortical neurons from E21 Sprague Dawley rats were cultured as described [26], using growth medium comprised of Neurobasal A medium + B27 (Invitrogen), 1% rat serum, 1 mM glutamine. Experiments were performed after a culturing period of 9–10 days during which cortical neurons develop a rich network of processes, express functional NMDA-type and AMPA/kainate-type glutamate receptors, and form synaptic contacts [29], [30]. Stimulations were performed after transferring neurons into defined medium lacking trophic support “TMo” [31]: 10% MEM (Invitrogen), 90% Salt-Glucose-Glycine (SGG) medium ([32]; SGG: 114 mM NaCl, 0.219% NaHCO_3_, 5.292 mM KCl, 1 mM MgCl_2_, 2 mM CaCl_2_, 10 mM HEPES, 1 mM Glycine, 30 mM Glucose, 0.5 mM sodium pyruvate, 0.1% Phenol Red; osmolarity 325 mosm/l, hereafter TMo). Stimulations were initiated approximately 48 h after transfection. HDAC inhibitors TSA (1 µM), Apicidin (0.5 µM) and Valproate (5 mM) were added for 8 h.

### Plasmids

GFP-SMRTα full length (GFP-SMRT^FL^) was a gift from Martin Privalsky (UC Davis; [18]). Plasmids GFP-SMRT^1–1523^, GFP-SMRT^Δ(1018–1523)^, and myc-SMRT^1025–1526^ have been described [16]. For the construction of GFP-SMRT^Δ(1523–1854)^ a MluI restriction site was inserted in GFP-SMRT^FL^ at position 1523 by site-directed mutagenesis and this construct was cut with MluI and BsrU36I blunt ended and re-ligated. The GFP-SMRT lacking the deacetylase activating domain (DAD) (GFP-SMRT^Δ(305–547)^) was made amplifying the whole GFP-SMRT^FL^ plasmid with the following primers: 5′-ata cgc gtc tgg tca tag cgc tgg ca -3′ and 5′-taa cgc gtg aca ctt ctg gcg agg aca ac-3′ which have a MluI restriction site, cut with MluI and re-ligate (adding to the sequence two extra amino acids, Thr and Arg). To clone myc-SMRT^1025–1861^ and myc-SMRT^1517–1861^ those regions were amplified using the following primers: myc-SMRT^1025–1861^ forward 5′-acg aat tca tgc cag tgc ctc ctg ccg aga aag ag-3′, reverse 5′-cgc tct aga tca cag atc ttc ttc aga aat aag ttt ttg ttc cgg gct gat ggg ctc cac ccc atc-3′, myc-SMRT^1517-1861^ forward 5-acg aat tca tgg acc acg ggg cac cct tca cca gt-3′, reverse 5′-cgc tct aga tca cag atc ttc ttc aga aat aag ttt ttg ttc cga aga ggt gga ggt gga cct-3′. PCR products were cloned in EcoRI/XbaI sites of pEF1/V5-His A expression vector (Invitrogen). Site-directed mutations were performed with the QuikChange II XL site-directed mutagenesis kit (Stratagene), following the manufacturer's instructions. Phenylalanine 451 within the DAD domain of SMRT was mutated to Alanine (SMRT^F451A^) using the following oligonucleotide and its reverse-complement: 5′-cac cct aag aac **gcc** ggc ctg att gcc-3′. An in silico NES search [33] revealed a single rodent/human conserved potential site IQELELRSL; aminoacids 1985 to 19993) in the C-terminus which contains the common LxxLxL motif (where L is L/I/V/F/M). Leucines 1988 and 1990 were mutated to Alanines (GFP-SMRT^ΔLeu^) with oligonucleotide 5′-ctt ttc cat cca gga **agc** gga **ag**c ccg ttc tct ggg tta cc-3′ and its reverse-complement.

### Transfections and immunofluorescence

Neurons were transfected using Lipofectamine 2000 (Promega) following the manufacturers instructions. Transfections were performed in TMo medium (see above) and carried out on primary neurons plated in 24 well plates. For each well, 2.67 µl of lipofectamine and 0.65 µg of plasmid DNA was used. siRNA directed against HDAC3 (Santa Cruz sc-270161) or control siRNA (Dharmacon's control non-targeting siRNA #2 siRNA) was used at 100 nM. Experiments were performed 48 h after transfection (72 h for experiments involving siRNA). Immunofluorescence was performed as described [34]. Anti-GFP antibody (1∶700; Invitrogen), anti-myc (1∶1000; Santa Cruz) and anti-HDAC3 (1∶300, Genetex) were used and visualized using biotinylated secondary antibody/cy2-conjugated streptavidin. Nuclei were counter-stained with DAPI. Pictures of GFP-SMRT-transfected neurons were taken on a Leica AF6000 LX imaging system, with a DFC350 FX digital camera. The DFC350 FX digital camera is a monochrome camera, and so coloured images essentially involve taking a black and white image (using the appropriate filter set) and applying a colour to the image after capture. Subcellular distribution of SMRT was scored as being either nuclear or having significant cytoplasmic localization (cytoplasmic distribution in the cell body of a equal or greater intensity than the nucleus). For each treatment, approximately 150–200 cells were analysed within 3–5 independent experiments.

### Statistical analysis

Statistical testing involved a 2-tailed paired student T-test. For any multiple comparisons within data sets we used a one-way ANOVA followed by Fisher's LSD post-hoc test.

## Results

### Inhibition of class I HDAC activity, likely HDAC3, is sufficient to promote SMRT export

**Figure 1:**
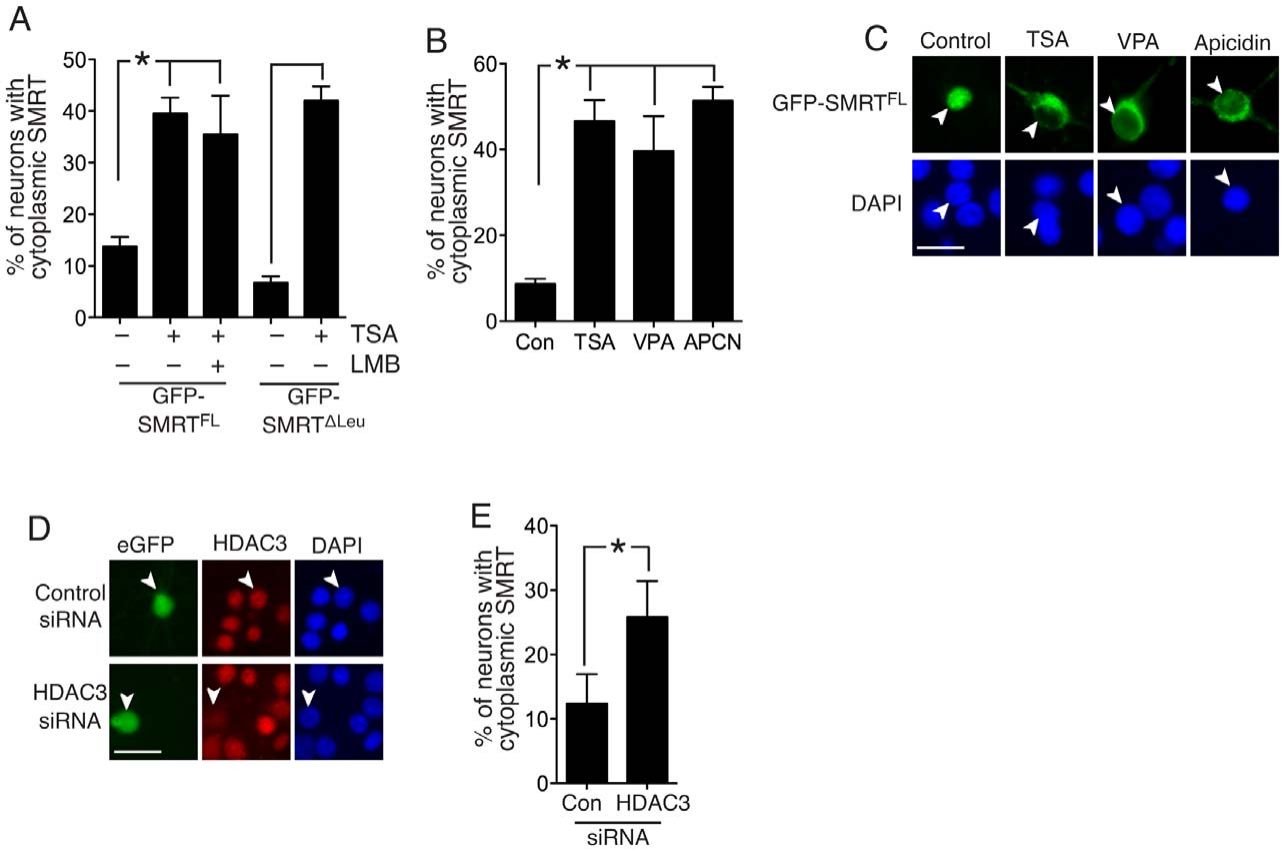
Inhibition of class I HDAC and knockdown of HDAC3 is sufficient to promote SMRT export. **A**) GFP-SMRT^FL^ export is insensitive to Leptomycin B and to mutation of a leucine-rich potential nuclear export sequence. Neurons were transfected with plasmids encoding GFP-SMRT^FL^ or GFP-SMRT^ΔLeu^ and treated, where indicated with Leptomycin B (20 ng/ml) for 1 h prior to treatment with TSA. Note that although redistribution was observed after 1–2 h treatment with TSA and other HDAC inhibitors, the effect observed was greater after 6–8 h and so all data presented in this manuscript relates to treatment with the indicated drugs for this time. *p<0.05 (n = 4). B) Neurons were transfected with plasmids encoding GFP-SMRT^FL^ and 48 h after transfection the neurons were treated with TSA, VPA or Apicidin and the cellular localization of GFP-SMRT^FL^ was analyzed. *p<0.05 (n = 3). C) Examples of the cellular localization of GFP-SMRT^FL^ after treatments with the indicated HDAC inhibitors. Scale bar is 20 µm here and throughout the manuscript. D) Example pictures to demonstrate the efficacy of HDAC3-directed siRNA in knocking down endogenous HDAC3 expression in rat cortical neurons. Neurons were transfected with the siRNAs as indicated, plus peGFP to identify transfected cells. After 72 h, cells were fixed and HDAC3 expression analysed by immunofluorescence. White arrows point to transfected neurons. E) HDAC3 siRNA causes redistribution of SMRT to the cytoplasm. Neurons were transfected with plasmids encoding GFP-SMRT^FL^ plus siRNA as indicated. SMRT localization was studied 72 h post-transfection. *p<0.05 (n = 5).

We previously reported that HDAC inhibition achieved by treatment with TSA promotes nuclear export of SMRT [26], prompting us to further investigate the mechanism and basis for this export. We confirmed our previous observation that treatment of cortical neurons with TSA caused the export of full length GFP-SMRTα (GFP-SMRT^FL^, Fig. 1a). Many proteins are exported via a CRM1-dependent association with a leucine-rich nuclear export site (NES), although many are not [35]. Search for a classical leucine-rich nuclear export site [33] revealed only one potential site at position 1985 (QELELRSL), which when mutated to QE**A**E**A**RSL (GFP-SMRT^ΔLeu^), had no effect on TSA-induced export (Fig. 1a), or indeed, export by synaptic activity (data not shown). Moreover, TSA-induced SMRT export was found to be insensitive to leptomycin B (Fig. 1a), and thus joins a lengthening list of proteins (that include many nuclear hormone receptors) whose export is independent of the CRM1/leucine-rich NES pathway.

SMRT has been reported to interact with the class I HDACs HDAC1 and HDAC3, and the class II HDACs HDAC4, HDAC5 and HDAC7 [1], [2], [3], [6], [11], [12]. Since TSA inhibits both Class I and II HDACs, we investigated whether TSA-induced export is due to Class I or Class II HDAC inhibition. We transfected neurons with a vector encoding GFP-SMRT^FL^ and after 48 h treated them with sodium valproate (VPA), a Class I-specific HDAC inhibitor [36]. VPA treatment was sufficient to induce GFP-SMRT export (Fig. 1b,c). We also found that treatment of neurons with apicidin, a HDAC inhibitor selective for Class I HDAC members HDAC2 and HDAC3 [36], was sufficient to promote GFP-SMRT export (Fig. 1b,c). Since HDAC3, but not HDAC2, interacts with SMRT and is a central part of SMRT and N-CoR corepressor complexes [3], [37], these pharmacological inhibition experiments suggest that a key mediator of TSA-induced SMRT export is the inhibition of HDAC3 activity. To test this directly we knocked down HDAC3 expression using siRNA (Fig. 1d). HDAC3 knockdown caused significant redistribution of SMRT to the cytoplasm, compared to a control siRNA (Fig. 1e). Taken together, these data indicate a role for HDAC3 activity in maintaining SMRT nuclear localization.

### Deletion or mutation of SMRT's DAD partly mimics the effect of HDAC inhibition

**Figure 2:**
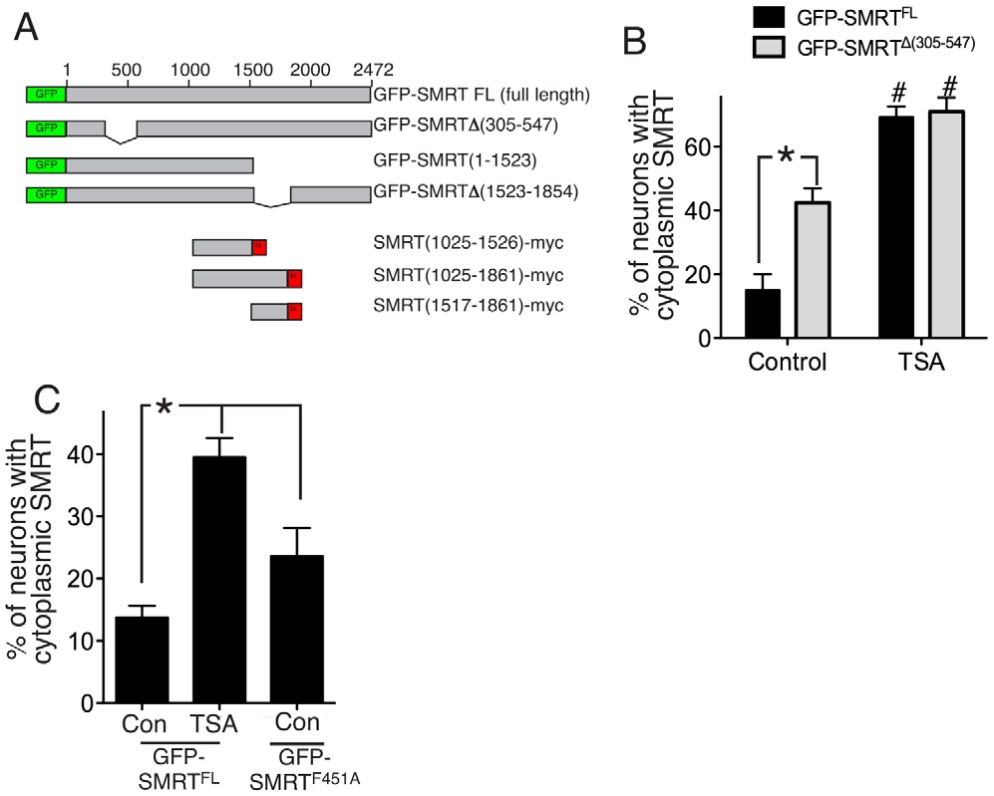
Deletion of SMRT's HDAC3-activating domain partly mimics and occludes the effect of HDAC inhibition. A) Schematic illustrating the SMRT deletion constructs generated and used in this paper. B) Analysis of the cellular localization of GFP-SMRT^FL^ or GFP-SMRT^Δ(305–547)^ in transfected neurons untreated or treated with TSA. *p<0.05 (n = 4). #*p*<0.05 comparing control and TSA-treated conditions for each SMRT construct. C) Analysis of the cellular location of GFP-SMRT^FL^ compared to the basal localization of GFP-SMRT^F451A^. *p<0.05 (n = 5).

HDAC3 forms a core complex with SMRT and is absolutely required for its function as a corepressor [6], [37]. HDAC3 has been reported to interact with SMRT at least two different regions, including the RD4 region [2] and also a region in the N-terminus referred to as the deacetylase activation domain (DAD). Interaction of HDAC3 with the DAD is both necessary and sufficient to activate the deacetylase activity of HDAC3, which is otherwise inactive [14]. As such, HDAC3 activity is restricted to complexes with SMRT or its close relative N-CoR. We therefore predicted that deletion of a portion of SMRT containing the DAD could, by inactivating HDAC3, mimic the effect of TSA treatment in promoting SMRT export. We deleted amino acids 305–547 within the context of full length 2472 amino-acid SMRT (GFP-SMRT^Δ(305–547)^), a schematic illustration of this and all SMRT constructs used in this study is shown in Fig. 2a. GFP-SMRT^Δ(305–547)^ exhibited increased cytoplasmic localization compared to GFP-SMRT^FL^ (Fig. 2b). The effect of DAD deletion in causing cytoplasmic redistribution was non-additive to the effect of TSA: TSA treatment of GFP-SMRT^Δ(305–547)^-expressing neurons caused a small additional export, but the total level of cytoplasmic SMRT was the same in TSA-treated neurons expressing GFP-SMRT^FL^ as GFP-SMRT^Δ(305–547)^. Given that deletion of DAD blocks SMRT-associated HDAC3 activity [14], this indicates that TSA is acting (at least in part) by blocking SMRT-associated HDAC3 activity. Since point mutation of the DAD at several locations can also inhibit DAD function and SMRT-associated HDAC3 activity [14], we created a DAD-inactivating mutant (GFP-SMRT^F451A^, [14]). As with GFP-SMRT^Δ(305–547)^, GFP-SMRT^F451A^ exhibited increased cytoplasmic localization compared to GFP-SMRT^FL^ (Fig. 2c), further evidence that DAD-induced HDAC3 activity is important for SMRT nuclear localization. Note though that deletion of the DAD, or its mutation did not completely mimic the effect of TSA treatment, potentially indicating that HDAC activity other than HDAC3 activated by the DAD may contribute to SMRT nuclear localization. Alternatively, since SMRT can homodimerize [38], dimerization between SMRT^Δ(305–547)^ or SMRT^F451A^ and endogenous SMRT could result in recruitment of active HDAC3 to the dimer.

### The RD4 region is necessary to mediate SMRT export following HDAC inhibition

**Figure 3:**
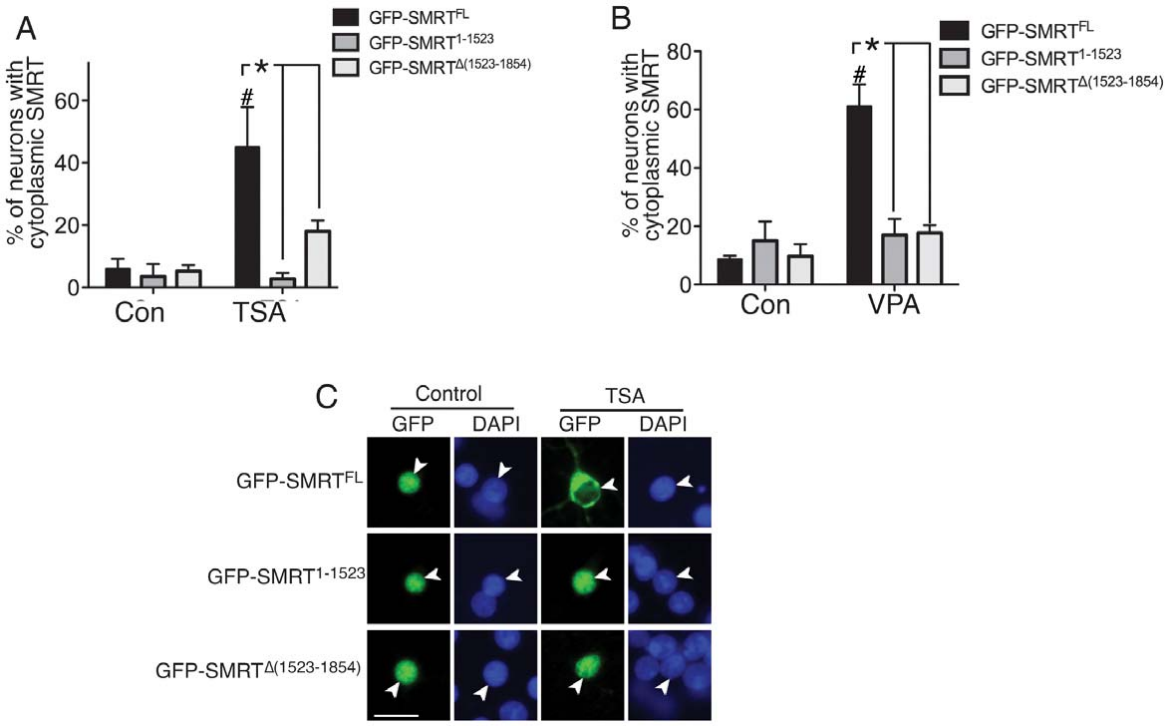
The RD4 region is necessary to mediate SMRT export following HDAC inhibition. Analysis of the cellular localization of the indicated GFP-SMRT fusions, transfected into neurons and treated with TSA (A) or VPA (B). *p<0.05 (n = 3). #*p*<0.05 comparing control and drug-treated conditions for each SMRT construct. C) Example pictures from (A). Scale bar 20 µm.

We next sought to identify the region of SMRT responsible for mediating nuclear export following HDAC inhibition. We found that truncating the C-terminus of SMRT from position 1524 onwards (GFP-SMRT^1–1523^) had no effect on basal nuclear localization of SMRT but completely abolished export following treatment with TSA, or VPA (Fig. 3a–c). Furthermore, the deletion of SMRT's RD4 region achieved by removing amino acids 1523–1854 (GFP-SMRT^Δ(1523–1854)^) also largely abolished export following treatment with TSA or VPA, without affecting basal nuclear localization (Fig. 3a–c). This indicated that the RD4 region may recruit factor(s) responsible for mediating CRM1-independent SMRT export upon HDAC inhibition.

**Figure 4:**
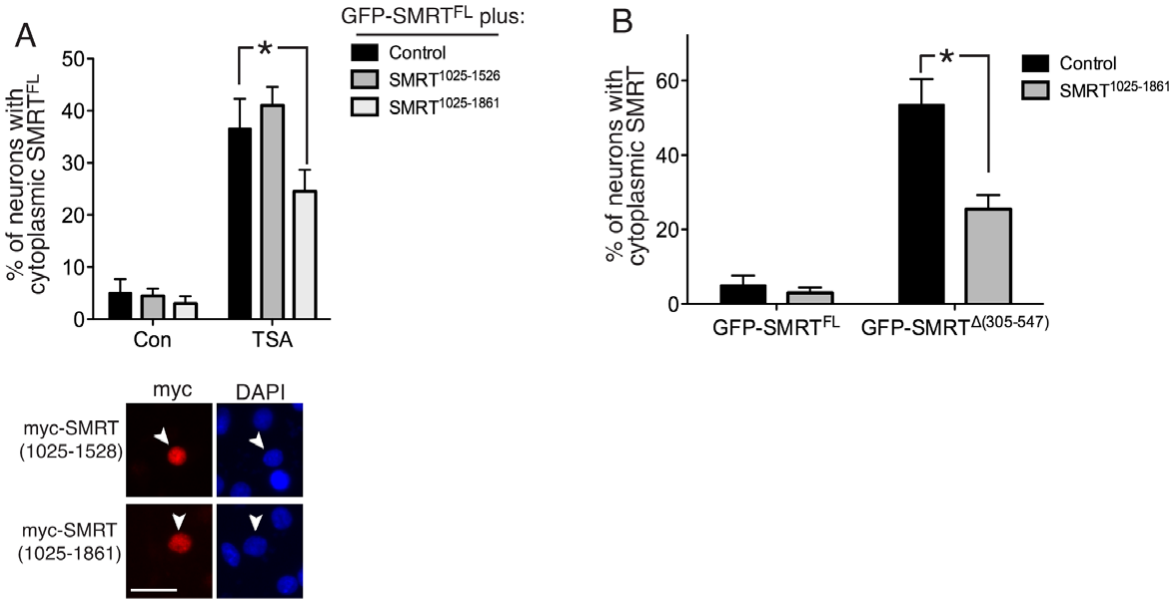
Over-expression of the RD4 region of SMRT inhibits HDAC inhibition-mediated SMRT export. A) *Upper:* Over-expression of RD4 inhibits TSA-mediated nuclear export of SMRT. Neurons were transfected with GFP-SMRT^FL^ plus an expression vector encoding the amino-acids 1025–1526 (RD3) or 1025–1861 (RD3–4) of SMRT or a control plasmid (encoding ß-globin). After 48 h neurons were treated with TSA and the subcellular localization of GFP-SMRT^FL^ was analyzed. *p<0.05 (n = 4). *Lower:* Example pictures to illustrate the nuclear localization of SMRT^1025–1526^ and SMRT^1025–1861^. Neurons were transfected with plasmids encoding myc-tagged SMRT^1025–1526^ or SMRT^1025–1861^. After 48 h the localization of these portions of SMRT was analysed by immunofluorescence using an anti-myc antibody. Arrows point to a transfected cell in each case. B) Over-expression of the RD4 region partially reverses the cytoplasmic redistribution of SMRT caused by deletion of the DAD. Neurons were co-transfected with GFP-SMRT^FL^ or SMRT^Δ(305–547)^ with an expression vector encoding the amino acids 1025–1861 of SMRT or a control plasmid (encoding ß-globin) as indicated. After 48 h the cellular localization of SMRT and SMRT^Δ(305–547)^ was analyzed. *p<0.05 (n = 3).

If this were indeed the case, we predicted that over-expression of the RD4 region within the nucleus would compete with SMRT for these hypothetical factors and inhibit TSA-induced export of GFP-SMRT^FL^. We first expressed a portion of SMRT containing the RD4 region (SMRT^1517–1861^) but found it to be exclusively cytoplasmic (data not shown), consistent with our recent observations that sequences N-terminal of position 1523 are required for nuclear localization of SMRT [16]. We therefore expressed a larger portion of SMRT (SMRT^1025–1861^), still including the RD4 region but with additional N-terminal sequence within the RD3 region which, upon expression, revealed nuclear localization (Fig. 4a lower). We therefore investigated the effect of expressing SMRT^1025–1861^ on TSA-induced export of GFP-SMRT^FL^. We found that co-expression of SMRT^1025–1861^ inhibited TSA-induced export of GFP-SMRT^FL^ (Fig. 4a, upper). To determine whether this effect could be attributed directly to the RD4 region (SMRT^1523–1861^), we investigated the effect of expressing SMRT^1025–1523^, the region N-terminal of the RD4 region (containing RD3) that we added in order to confer nuclear localization. Expression of SMRT^1025–1523^, which is localized to the nucleus ([16] and Fig. 4a lower), failed to inhibit TSA-induced export of SMRT^FL^ (Fig. 4a, upper), strongly indicating that the inhibitory effect of SMRT^1025–1861^ is due to the presence of the RD4 region and not the RD3 region. Thus, over-expression of RD4 region interferes with TSA-induced SMRT^FL^ export, consistent with the deletion studies and further implicating this domain as being required for interaction with the SMRT export machinery.

Given that deletion of a region of SMRT containing the DAD (SMRT^Δ(305–547)^) results in partial redistribution of SMRT to the cytoplasm, we reasoned that co-expression of SMRT^1025–1861^, by interfering with the export process, might reduce the cytoplasmic localization of SMRT^Δ(305–547)^. This was indeed found to be the case: the proportion of neurons with cytoplasmic SMRT^Δ(305–547)^ was substantially reduced by co-expression of SMRT^1025–1861^ (Fig. 4b). In contrast expression of SMRT^1025–1523^ had no effect (data not shown). Thus, over-expressing the SMRT RD4 region partially reversed the effect of deleting the DAD with respect to SMRT nuclear localization.

## Discussion

Here we have presented data which suggests that class I HDAC activity is necessary for nuclear retention of the corepressor SMRT in neurons. HDAC3, a known component of the core SMRT complex, is likely to be an important HDAC responsible for SMRT nuclear retention as suggested by the fact that the specific HDAC2/3 inhibitor Apicidin promotes SMRT nuclear export, as does HDAC3 knock-down, as well as deletion and mutation of the HDAC3-activating DAD region. Additionally, deletion and over-expression studies implicate the RD4 region as a key mediator of HDAC inhibitor-induced SMRT export.

### Lysine acetylation controls protein interactions and subcellular localisation

In recent years lysine acetylation of non-histone proteins has emerged as an important post-translational protein modification regulating function in different ways including protein interactions and subcellular localization [39]. Global acetylome analysis in three different human cell lines identified 3600 lysine acetylation sites on 1750 proteins, 17 of those proteins have a function in nuclear transport, suggesting that this process is sensitive to the cells acetylase-deacetylase balance [40]. Furthermore, many transcription factors have been shown to move between the cytoplasm and nucleus in a manner dependent on their direct acetylation.

For example, p53 acetylation by p300 mediates its nuclear export [41], while SIRT1/2-mediated deacetylation promotes FOXO1's nuclear localization [42], [43]. Acetylation of Poly(A)-polymerase disrupts its interaction with Importin-a/b complex resulting in cytosolic accumulation [44]. Several transcription factors involved in development exhibit acetylation-sensitive subcellular localization. In embryonic stem cells, acetylation of Sox2, a factor important for maintenance of pluripotency, induces its nuclear export [45]. Acetylation of SRY, (crucial for testis organogenesis) by p300 augments its nuclear import, while its deacetylation by HDAC3 induces a partial cytoplasmic distribution [46].

Of note, SMRT is also involved in development, particularly neurogenesis where it is needed to provide fidelity to both Notch- and retinoic acid-dependent aspects of forebrain development and neurogenesis [15]. Despite our investigations into the role of acetylation in SMRT export, it remains unclear whether SMRT redistribution is due to direct changes in its own acetylation. Human SMRT was reported to have least three acetylatable lysine residues at positions 959, 1794 and 2036 [40]. Given the sensitivity of SMRT localization to HDAC inhibition we mutated these residues to non acetylatable arginine residues. However, mutation of these sites alone or in triple combination did not prevent the TSA-mediated SMRT export (data not shown). Thus, it remains unclear whether HDAC inhibition-mediated SMRT export is a consequence of a direct modification in the acetylation status of SMRT or a modification in its associated proteins in the SMRT complex, or indeed in proteins involved in SMRT export.

### HDAC3 is important for full nuclear localization of SMRT

Although other HDACs may contribute, data presented in this study indicates that HDAC3 is likely to be an important deacetylase responsible for retaining SMRT in the nucleus. HDAC3 absolutely requires SMRT's DAD domain for HDAC activity and thus acts as a coenzyme of HDAC3 [14]. In our study, SMRT mutants either lacking the DAD domain (GFP-SMRT^Δ(305–547)^), or with a DAD-inactivating mutation (GFP-SMRT^F451A^), partially mimicked the effect of HDAC inhibitors in promoting cytoplasmic localization, as did HDAC3 knockdown. Nonetheless, this GFP-SMRT^Δ(305–547)^ was further exported in presence of HDAC inhibitors which suggest that HDACs other than HDAC3 may contribute to SMRT nuclear localisation. Alternatively, since SMRT can homodimerize [38], dimerization between SMRT lacking the DAD with wild-type SMRT may recruit active HDAC3 to the complex, thus explaining the partial effect of DAD deletion. As stated earlier, disruption to the DAD of SMRT or N-CoR essentially abolished both HDAC3 activity and corepressor function [14], [47]. The fact that this also causes some cytoplasmic redistribution of SMRT (this study) raises the possibility that elimination of repressor function could be due in part to SMRT relocalization to the cytoplasm. While this is a possibility, the effect of DAD disruption on SMRT corepressor function is more dramatic than its effect on nuclear localization ([14] and this study), indicating that even nuclear-localized SMRT is inactive if it lacks a functional DAD. Thus the conclusions of the earlier studies by Lazar and coworkers are not in question.

### A model to explain histone deacetylase-dependent SMRT localization

In contrast to deletion/mutation of the DAD, which caused basal cytoplasmic redistribution, deletion of the RD4 region had no effect on basal localization but strongly reduced export triggered by HDAC inhibition. Thus the HDAC3-suppressed export process acts on SMRT in a manner that requires the RD4 region. This could be explained by two potential models. Export machinery could be recruited to the RD4 region of SMRT but in a manner that requires acetylation within this region, an event that is ordinarily suppressed by HDAC3. As a variation on this, the export machinery recruited to the RD4 region of SMRT could itself be directly or indirectly inhibited by HDAC3 activity. Distinguishing between these models forms part of ongoing investigations. Regardless of this, it is clear that SMRT export triggered by HDAC inhibition is mechanistically distinct from another form of signal-induced SMRT export-that induced by synaptic activity [16], [26]. In contrast to results presented here, deletion of the RD4 region has no effect on SMRT export triggered by synaptic activity [16].

### Inhibition of HDACs as a neuroprotective strategy

In recent years the use of HDAC inhibitors has emerged as a potential therapy against cancer and neurodegenerative disorders, including Parkinson's, Alzheimer's, Huntington's diseases and amyotrophic lateral sclerosis. Beneficial effects of HDAC inhibition have been found in models of the above diseases, as well as in acute trauma such as stroke [27]–[28]. Given the growing number of non-histone HDAC targets been discovered, old assumptions based on mechanisms solely surrounding histone acetylation status are being challenged [48]. Moreover, the pleiotropic effects of HDAC inhibition and neurotoxicity of sustained strong inhibition raise the question as to their suitability for treating chronic neurodegenerative disease [49], particularly given the documented adverse side-effects of HDAC inhibitors after short-term therapy in cancer patients [50]. In order to find more selective and tolerable inhibitors of HDAC effects, future strategies may be to use peptides or molecules designed to disrupt interactions between HDACs and key targets relevant to neuroprotection. We recently demonstrated a neuroprotective consequence of promoting SMRT export, since when nuclear it is able to antagonize the neuroprotective, antioxidant effects of the transcriptional coactivator PGC-1α [16]. Further investigation into the mechanism of SMRT export may point to strategies aimed at controlling SMRT-mediated repression.
